# The fecal microbiota from children with autism impact gut metabolism and learning and memory abilities of honeybees

**DOI:** 10.3389/fmicb.2023.1278162

**Published:** 2023-11-23

**Authors:** Yiyuan Li, Yan Zhang, Xi Luo, Yujie Meng, Zhaopeng Zhong, Hao Zheng, Yunsheng Yang

**Affiliations:** ^1^Microbiota Division, Department of Gastroenterology and Hepatology, First Medical Center, Chinese PLA General Hospital, Beijing, China; ^2^Medical School of Chinese PLA, Beijing, China; ^3^College of Food Science and Nutritional Engineering, China Agricultural University, Beijing, China

**Keywords:** *Apis mellifera*, autism, human fecal microbiota, cognitive impairment, microbiota-gut-brain axis

## Abstract

Autism spectrum disorder (ASD) is a set of neurodevelopmental disorders, with an increasing incidence. Gastrointestinal symptoms are common comorbidities of ASD. The gut microbiota composition of children with autism is distinct from that of typical developmental (TD) children, suggesting that the gut microbiota probably influences on hosts via the microbiota-gut-brain axis. However, the relationship between intestinal dysbiosis and host brain function remains unclear. In this study, we creatively developed a honeybee model and investigated the potential effects of fecal microbiota on hosts. Fecal microbiota from children with autism and TD children were transplanted into microbiota-free honeybees (*Apis mellifera*), resulting in induced ASD-fecal microbiota transplantation (FMT) honeybees (A-BEE group) and TD-FMT honeybees (T-BEE group), respectively. We found that cognitive abilities of honeybees in the A-BEE group were significantly impaired in olfactory proboscis extension response conditioning. Metagenomics was used to evaluate fecal microbiota colonization, revealing several differential species responsible for altered tryptophan metabolism and taurine metabolism within the bee gut, including *Bacteroides dorei*, *Bacteroides fragilis*, *Lactobacillus gasseri*, and *Lactobacillus paragasseri*. Furthermore, fecal microbiota from children with autism downregulated brain genes involved in neural signaling and synaptic transmission within honeybees. Notably, differentially spliced genes observed within brains of honeybees from the A-BEE group largely overlapped with those identified in human diagnosed with autism via SFARI and SPARK gene sets. These differentially spliced genes were also enriched within pathways related to neural synaptic transmission. Our findings provide novel insights into the pivotal role of the human gut microbiota, which may contribute to neurological processes in honeybees. Additionally, we present a few research sources on gut-brain connections in ASD.

## 1 Introduction

Honeybees (*Apis mellifera*), as social insects, exhibit complex social behaviors and brain functions. Moreover, their stable and simple gut microorganisms provide certain availability for investigating the microbiota-gut-brain interaction (Engel et al., 2012; Zheng et al., 2018; Zhang et al., 2022b). The research conducted by our team revealed that the gut bacteria of bees not only influence their behavior and metabolism in the intestines but also modify the levels of neurotransmitters and the transcriptional programs in their brains (Zhang et al., 2022a,b). *Gilliamella* regulates carbohydrate and glycerophospholipid intestinal metabolism pathways (Zheng et al., 2016), whereas *Lactobacillus* Firm4 and Firm5 primarily affect amino acid metabolism pathways, thereby influencing circulating metabolites and cognitive behavior (Zhang et al., 2022a). Interestingly, our research has found unique gene expression characteristics in the transcriptome of behaviorally disrupted bee brains that are associated with genes involved in regulating olfactory function and labor division. These bees usually do not respond to social stimuli (Zhang et al., 2022a). Notably, there is evidence of homologous molecular mechanisms underlying social responses between bees and humans (Shpigler et al., 2017). Psychiatric disorders have also been linked to atypical alternative splicing (AS) of genes related to autism spectrum disorder (ASD; Sharon et al., 2019). Furthermore, we have identified overlapping AS genes associated with behavioral disruption in bee brains that coincide with human autism gene data sets (Zhang et al., 2022b). The brains of mice colonized by the gut microbiota from human with autism are also affected (Sharon et al., 2019). In an autism mouse model study, Golubeva et al. found that the interactions between intestinal microbiota, gut physiology, and social behavior (Golubeva et al., 2017). These findings suggest that deep conservation of genes related to social responsiveness across humans and distant insect species provides an opportunity to investigate microbiota-gut-brain interaction. Bees transplanted with human fecal microbiota may play a helpful role in exploring the relationship between autism and intestinal microbiota.

ASD is a set of heterogeneous neurodevelopmental disorders characterized by social interaction disorders, language communication disorders, and repetitive stereotypical behavior, that seriously impact the lives of those with autism and place heavy psychological and economic burdens on their families and society (Lai et al., 2014). Autism has a large male predominance with a male to female ratio of approximately 4:1 (Wang J. et al., 2022). The prevalence of ASD has increased 20–30 fold in the last 40 years globally, but the causes are not well-understood (Wang J. et al., 2022). Rare gene changes, or mutations, as well as small common genetic variations have been detected in subjects with autism, implying a genetic component (Fang et al., 2021, 2023). A growing area of research has focused on the interaction of genetic and environmental factors, and the gut microbiota represents an interface between environmental and genetic risk factor (Waltes et al., 2019; Chen et al., 2020; Yu et al., 2023). Gastrointestinal symptoms are common comorbidities of ASD (Lai et al., 2014; Vuong and Hsiao, 2017), suggesting that microbiota dysbiosis is involved in ASD (Finegold et al., 2010; Adams et al., 2011; Vuong and Hsiao, 2017; Kang et al., 2018; Wan et al., 2022). A study on the gut microbiota found that compared with typical developmental (TD) children, there were differences in both alpha and beta diversities of gut microbes in children with autism (Liu et al., 2019). In addition, intestinal metabolomics revealed differences in the intestines of children with autism, along with differences in the microbiota. The study found that compared with healthy individuals, intestinal metabolism in individuals with autism differed in multiple microorganism-mediated pathways, including bile acid metabolism, taurine metabolism, unsaturated fatty acid metabolism, and tryptophan metabolism (Agus et al., 2018; Zhu et al., 2022). In a study comparing metabolites in the urine of children with autism and TD children, one of the most differentiated metabolic pathways was that of tryptophan. In children with autism, the kynurenine pathway was upregulated and the serotonin pathway was downregulated, resulting in melatonin deficiency (Gevi et al., 2016). A clinical study found that sleep disorders in children with autism were associated with cognitive decline, bad mood, and behavioral disorders, and that appropriate and regular melatonin supplementation could relieve these symptoms (Galli et al., 2022). These differential metabolites are often essential for the central nervous system’s growth, development, and normal functioning (Evans et al., 2008). Moreover, studies have reported that fecal microbiota transplantation (FMT) can improve Tourette syndrome and autism symptoms (Kang et al., 2017, 2019; Zhao et al., 2017, 2020; Li N. et al., 2021; Nirmalkar et al., 2022). Our team performed FMT to treat six children with autism, producing significant improvements in their symptoms. These findings were published in a case report in 2017 (Huijun Zhao et al., 2017). These findings suggest that gastrointestinal microbes affect immune, metabolic, and nervous system development and function in the host. Studies on gut microbiology may be an important avenue for understanding autism.

Animal models commonly employed in the study of ASD diseases aim to replicate the core symptoms associated with ASD, particularly late-stage brain dysfunction such as social, learning, and emotional impairments. Honeybees are social insects, possessing a unique behavioral structure characterized by a series of complex social interactions within and beyond the hive. These complex behaviors largely depend on sensory sensitivity, particularly olfactory sensitivity (Braun and Bicker, 1992; Moauro et al., 2018; Leger and McFrederick, 2020). For instance, bees can use olfactory information to locate food sources, recognize nestmates and communicate information, identify potential predators to resist harm, transmit hormonal signals and engage in feeding behavior (Arenas et al., 2007; Farina et al., 2007; Sandoz, 2011). Behavioral protocols have been established to quantitatively evaluate the learning and memory abilities of honeybees based on their olfactory proboscis extension response (PER), and we adopted this method as an indicator for studying bee brain function (Sandoz, 2011; Matsumoto et al., 2012; Van Nest, 2018; Zheng et al., 2018). Moreover, microbiota-free animals have unique advantages when studying the mechanisms of diseases related to the microbiota-gut-brain axis (Bauman and Schumann, 2018; Li Z. et al., 2021). The use of microbiota-free honeybees is a well-established technique that obviates the need for antibiotic administration, thereby closely resembling the organism’s natural physiological state (Zheng et al., 2018; Zhang et al., 2022a). Therefore, microbiota-free honeybees were selected for this study.

In this study, we attempted to transplant fecal microbiota from children with autism into the gut of microbiota-free bees to develop a new honeybee model for studying ASD. Behavioral experiments were used to assess the learning and memory abilities of honeybees. Metagenomics was used to evaluate the colonization of fecal microbiota in the intestines of bees receiving FMT, the in-depth exploration of the effect of ASD fecal microbiota on the gene expression characteristics of the bee brain transcriptome, and the impact of ASD fecal microbiota on intestinal metabolism. Thus, we attempted to validate the potential utility of bees as a novel model in ASD microbiome research, aiming to provide novel possible clues for understanding ASD mechanisms.

## 2 Materials and methods

### 2.1 Children clinical information collection and fecal sample collection

The ASD-CHILD group, consisting of children diagnosed with ASD, was recruited at the First Medical Center of Chinese PLA General Hospital. The normally developing children (the TD-CHILD group) were enlisted from the kindergartens in the identical vicinity. Consent was obtained from the guardians of all participants for the collection of fecal samples and clinical information. The caregivers/parents received brief training on the use of sterile medical containers and sterile tubes Subsequently, the caregivers/parents immediately transferred the fresh feces to an insulated box with ice cubes. The feces were delivered to the laboratory on ice within 2 h. Transportation of the fecal samples was handled by designated members of our team. Approval for the study was granted by the Ethics Committee of Chinese PLA General Hospital, with the assigned approval number S2015-110-02. The legal guardians of all children provided written informed consent before enrollment. Enrolled subjects were not allowed participate in another study.

Children with autism who met the following criteria were included: (1) ≥3 years old and diagnosed with ASD according to the Diagnostic and Statistical Manual of Mental Disorders, Fifth Edition criteria and Autism Diagnostic Observation Schedule, Second Edition (ADOS-2) and (2) experienced gastrointestinal symptoms (Schneider et al., 2006; Gotham et al., 2007; Jurek et al., 2022). The exclusion criteria for children with autism were as follows: (1) children diagnosed with fragile X syndrome, karyotype abnormalities, other genetic diseases, and/or brain malformation; (2) had ulcerative colitis, Crohn’s disease, or bowel cancer; (3) had undergone a probiotic/prebiotic intervention in the last 3 months; (4) underwent FMT treatment; (5) had a serious dietary preference; or (6) used antibiotics and antifungal medications 3 months before fecal sample collection. Children with autism were allowed to continue their routine treatments, such as rehabilitation training or psychiatric medications. The Childhood Autism Rating Scale, Second Edition (CARS-2) is applied to assess the overall severity of core ASD symptoms, and based on this scale, patients are characterized as follows: “not autism (<30),” “mild-to-moderate autism (30–36.5),” or “severe autism (>36.5)” (Chlebowski et al., 2010; Dawkins et al., 2016; Jurek et al., 2022). CARS-2 includes a 15-item scale (14 questions regarding discrete behaviors and 1 opinion question on autism), with each item having a score of 1–4 that reflects the severity of deviation compared to expectations for peers of the same chronological age. The TD children had to fulfill the subsequent conditions: (1) no ASD or developmental disorders, such as attention deficit hyperactivity disorder, anxiety, depression, oppositional defiance disorder or severe depressive disorder; (2) no first-degree relatives with a mental disorder; (3) no gastrointestinal symptoms in the past 3 months; and (4) no intake of probiotics or antibacterial agents in the past 3 months. The caregivers/parents of TD children completed a screening questionnaire about medical and family history, and the children underwent laboratory blood, urine, and stool examinations to dismiss the possibility of infectious diseases and metabolic syndromes. The detailed process of donor screening was based on the methodology previously established by our team (Wang et al., 2020; Zhao et al., 2020). The guardians of all children included in this study completed consultations about their children’s dietary habits. We assessed various dimensions that might affect nutritional intake, including ethnicity, food selectivity, pica, and quantity of food intake. The gastrointestinal symptoms of children were evaluated using the gastrointestinal severity index (GSI), including constipation, diarrhea, average stool consistency, stool smell, flatulence, abdominal pain, unexplained daytime irritability, nighttime awakening, and abdominal tenderness (Adams et al., 2011). Previous research indicated that gastrointestinal symptoms are common in children with autism, and such children tend to exhibit high risk of intestinal microbiota dysbiosis (Wang J. et al., 2022; Wang H. et al., 2023). Therefore, we recruited children with autism accompanied by gastrointestinal symptoms. The GSI was completed by caregivers/parents during each assessment. There are gender differences in children with autism (Wang J. et al., 2022). However, the honeybees in the experiment were worker bees, and all worker bees are female in natural world. Therefore, gender factors were not considered in the study.

### 2.2 Generation of microbiota-free honeybees

All honeybees (*Apis mellifera*) used in this study were obtained from honeycombs provided by the Dr. Zheng laboratory. Microbiota-free honeybees were generated in Dr. Zheng’s laboratory using Dr. Zheng’s methods (Zheng et al., 2019). During the transition from honeybee pupa to adult emergence, there is a natural microbiota-free period when newly emerged workers contain few or no bacteria (Engel et al., 2012; Zheng et al., 2018; Zhang et al., 2022b). Therefore, we referred to the honeybees obtained during this physiological stage as microbiota-free bees. Briefly, at the late stage of pupation, the pupae were manually transferred to sterile incubators where they continued to develop at 35°C and 50% humidity until eclosion. As the gut bacteria in honeybees are not strictly anaerobic, honeybee guts were ground and diluted to a cell count of 1 × 10^7^, and the absence of bacterial growth on agar plates was used to define microbiota-free honeybees. The newly emerged honeybees (Day 0) were placed in sterile cup cages and fed sterile sucrose solution and sterile pollen for 24 h, after which they were divided into three groups, namely conventional (CV group) honeybees, ASD-FMT honeybees (A-BEE group), and TD-FMT honeybees (T-BEE group), with 25–30 bees per cup, and subjected to the corresponding interventions (Day 1). The pollen and sucrose solution provided to each group of bees were identical to avoid the impact of differences in bee diet on the study.

### 2.3 Honeybee colonization

In their natural state, newly emerged honeybees primarily acquire a relatively stable gut microbiota through feeding for 3–4 days (Zheng et al., 2018). Therefore, this study conducted FMT on microbiota-free honeybees by adding human fecal microbiota to their feed. Fresh fecal samples from ASD-CHILD and TD-CHILD donors were collected promptly and transferred by their parents to the laboratory within 4 h, after which they were suspended in 25% sterilized glycerol and stored in a sample bank at −80°C. Frozen fecal samples were pretreated before honeybee colonization. Briefly, the fecal samples were resuspended in sterilized phosphate-buffered saline (PBS; Solarbio, Beijing, China) to a final concentration of 0.1 g/ml, and the larger food residues were filtered out through a sterile 100 μm diameter filter membrane (Biologix, Shandong, China). The filtrate was then centrifuged at 1,000 × *g* and 4°C for 3 min to extract the supernatant (Sharon et al., 2019). For the colonization of the A-BEE group, 1 ml of supernatant from ASD-CHILD fecal samples was collected as described above and mixed thoroughly with 1 ml of sterilized sucrose solution (50%, w/v) and 0.3 g of sterilized pollen to obtain a final fecal suspension concentration of 0.05 g/ml for feeding. For the colonization of the T-BEE group, food was prepared using the same method but using TD-CHILD fecal samples. The CV group was fed a mixture of 5 μl of wild bee hindgut homogenate with 1 ml of 1× PBS, 1 ml of sterilized sucrose solution (50%, w/v), and 0.3 g of sterilized pollen. Experimental honeybees were fed suspension-containing chow for the first 3 days (Days 1–3), with food changed every 24 h. Each honeybee group was then placed in an incubator at 35°C and 50% relative humidity and provided with sterilized sucrose (50%, w/v) and sterilized pollen until Day 8. On Day 8, behavioral experiments were conducted on each honeybee group. At the end of the experiment, the brains and intestines of the honeybees were collected for additional analyses.

### 2.4 Learning and memory test

**Figure 1:**
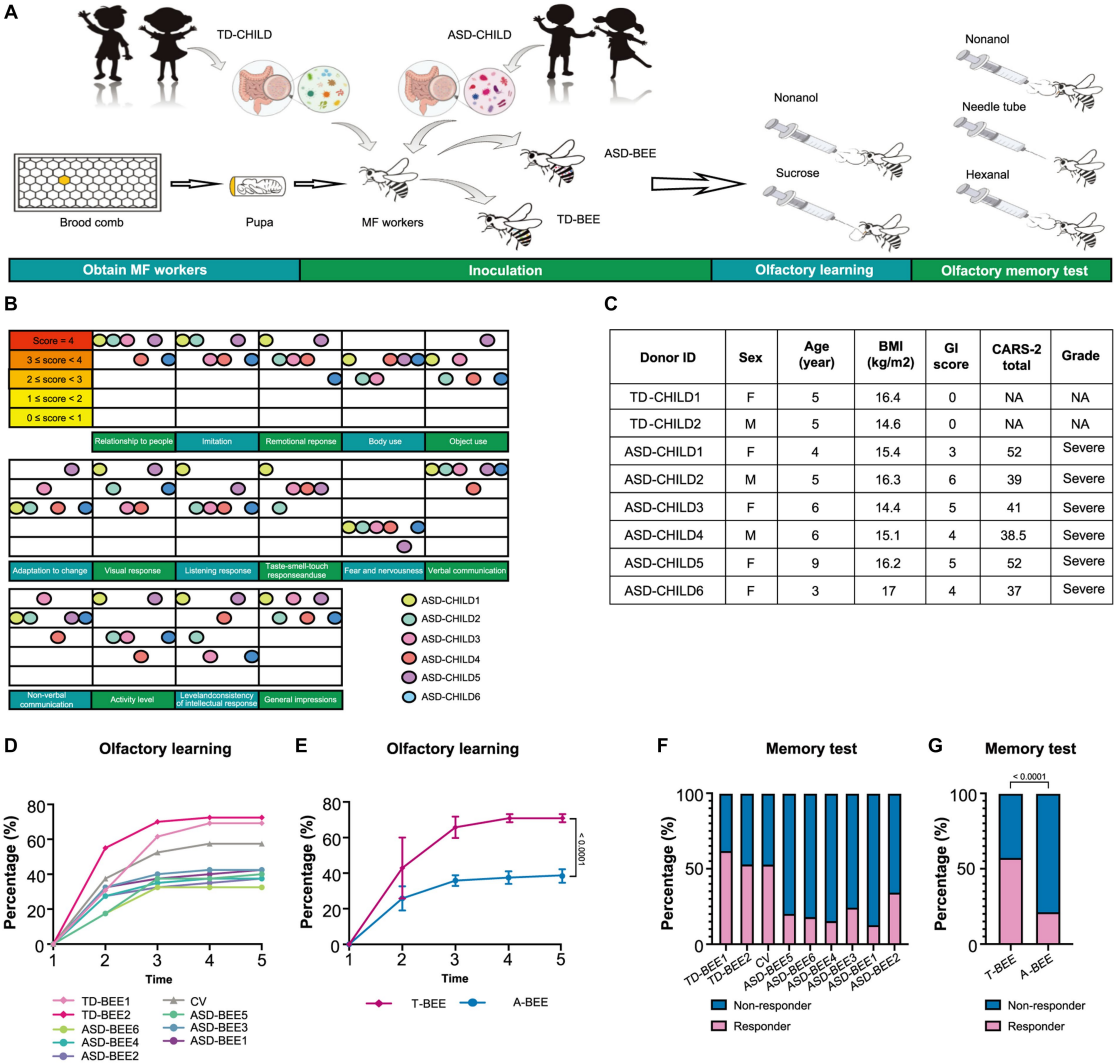
Fecal microbiota from ASD donors affects the learning and memory of honeybees. **(A)** Schematic illustration of the experimental design: microbiota-free honeybees were fed sterile sucrose water and pollen for the first 24 h after eclosion. Then honeybees aged 1–3 days were colonized with fecal microbiota from TD or ASD donors. Olfactory learning and memory experiments were performed when the honeybees were 8 days old, after which tissues and samples were collected. **(B)** ASD donors’ scores for the 15 items in CARS-2. The color of the ball represents each child with autism, and the position of the ball represents the child’s score on the corresponding item. **(C)** Basic clinical information of the donors (see Supplementary Table S1 for more details). **(D)** Learning curves for a positively rewarded conditioned stimulus [CS; sample size: TD-BEE 1 (*n* = 39), TD-BEE 2 (*n* = 40), ASD-BEE 1 (*n* = 40), ASD-BEE 2 (*n* = 40), ASD-BEE 3 (*n* = 40), ASD-BEE 4 (*n* = 40), ASD-BEE 5 (*n* = 40), ASD-BEE 6 (*n* = 40), CV (*n* = 40); *N* = 359 in total]. **(E)** Learning curves for the A-BEE group (includes ASD-BEE 1–6) and the T-BEE group (includes TD-BEE 1 and 2). Differences between groups were determined using the chi-squared test. **(F)** Ratio of honeybees that were successful in the memory test (sample sizes are the same as those in **D**). **(G)** Ratio of honeybees successful in the memory test in the A-BEE and the T-BEE groups. Differences between groups were determined using the chi-squared test.

Considering that the ability to recognize, understand, and remember odors is the cornerstone of many of the social activities in bees and one of the essential means of communication among individuals, we performed experiments based on olfactory learning and the PER to reflect changes in bee behavior and brain function (Sandoz, 2011; Huijun Zhao et al., 2017; Liu et al., 2017; Van Nest, 2018). The olfactory learning and memory abilities of each honeybee group were measured on Day 8 (i.e., when they were 8 days old). We used methods reported in previously published studies by our team (Chang et al., 2022; Zhang et al., 2022a). The honeybees were then placed in a rearing box for 2 h of starvation. In the test, nonanol (1-nonanol, purum ≥98.0% GC; Sigma-Aldrich, St. Louis, MO, USA) and n-hexanal (caproaldehyde, purum ≥99.0% GC; Macklin; Shanghai, China) were used as odor sources, with nonanol used for olfactory learning and n-hexanal used as a negative control. During conditioning, all honeybees were subjected to five rounds of training, with each round lasting 10 min. After five rounds of training, the honeybees were left in the experimental environment for 2 h without being fed. Subsequently, they were subjected to a memory test. Briefly, two odor stimuli (nonanol or hexanal) were presented randomly, and a clean odorless syringe was presented after each odor stimulus to exclude the effects of visual stimuli. PER was observed and recorded, and honeybees that performed PER only under nonanol stimulation were considered to have succeeded in the memory tests (Figure 1A).

### 2.5 Tissue collection

After completing the behavioral tests, the entire intestine of each honeybee, including the midgut, ileum, and rectum, was dissected on ice. The intestine was collected in a 1.5 mL sterile centrifuge tube and stored in liquid nitrogen in a freezer at −80°C. On the same day, the thorax was fixed to a beeswax plate using a dissecting needle, and the entire brain was obtained by removing the head cuticle under a dissecting microscope (Nikon SMZ745T, Japan). The whole honeybee brain was placed on a slide and immersed in RNAlater (Thermo Fisher Scientific, Waltham, MA, USA). In addition, the glands on the brain tissue (pharyngeal and salivary glands), two compound eyes, and three single eyes were removed on ice using cell forceps and placed in sterilized 1.5 ml centrifuge tubes that were stored in liquid nitrogen. These samples were also stored in a freezer at −80°C.

### 2.6 Hematoxylin–eosin staining of the intestines

The dissected honeybee intestines were fixed into a fixative solution (8% w/v paraformaldehyde), rinsed with PBS, dehydrated in ethanol, and transferred into xylene. The tissue was then placed in paraffin wax, which was dried at 60°C.The paraffin-embedded tissue was cooled to complete solidification, after which it was sectioned at a thickness of 4–6 mm. The sections were consecutively submerged in xylene and a series of ethanol solution, dyed with hematoxylin for 5 min, and processed with 1% acidic ethanol (1% hydrochloric acid in 70% ethanol). Then, the objects were washed in purified water, treated with eosin for 3 min, and submerged again in ethanol and xylene. A microscope (Leica, Wetzlar, Germany) was used to examine the morphological composition of the intestinal tissue.

### 2.7 Honeybee intestinal DNA extraction and shotgun metagenome sequencing

The entire digestive tract of the honeybee was pulverized using a sterile disposable mortar and pestle that was free of enzymes, and genomic DNA was extracted from each sample individually using cetyltrimethylammonium bromide (CTAB) bead beating. The specific experimental procedure details can be found in the team’s previous research (Chang et al., 2022). Six biological replicates of each group were used for metagenomic sequencing analysis.

The DNA samples were sent to BGI Genomics Co., Ltd. (Guangzhou, Shenzhen, China) for shotgun metagenome sequencing. Sequencing was conducted on a DNBSEQ-T1&T5 system, resulting in the generation of 150 bp paired-end reads. The sequencing quality was assessed using fastp v0.23.2 with its default parameters. Reads belonging to the honeybee reference genome (version Amel_HAv3.1) and human reference genome (version hg_19) were removed separately using BWA v0.7.17-r1188 and SAMtools v1.9. Metaphlan2 v3.0.14 was employed to analyze the community structure of each sample at the phylum and species levels, employing its default parameters. Then we ran the “merge_metaphlan_tables.py” script in Metaphlan2 to merge our human bacterial genome samples into one table in which the relative normalized abundances per sample were listed.

### 2.8 Honeybee brain RNA extraction and transcriptome sequencing

Six biological replicates were included in each group for transcriptome sequencing. Total RNA from the honeybee brain was extracted using a Quick-RNA MiniPrep Kit (Zymo Research, Irvine, CA, USA). The RNA samples from the honeybee brains were sent to BGI Genomics Co., Ltd. (Guangzhou, Shenzhen, China) for transcriptome sequencing. The sequencing was conducted on a DNBSEQ-T1&T5 system, resulting in the generation of 150 bp paired-end reads.

Fastp v0.23.2 was used to evaluate the sequencing quality of each sample, employing its default parameters. Using HISAT2 v2.2.1, we constructed an index of the honeybee reference genome (Amel_HAv3.1) and aligned the fastp-trimmed reads to this index using HISAT2 with default parameters. Quantification of gene expression was performed using HTSeq v2.0.1 in the “union” mode, considering only reads that clearly mapped to a single gene. Reads that were aligned to multiple positions or overlapped with more than one gene were excluded. Differential expression analysis was performed using the DESeq2 package (v1.34.0) in R (v4.1.0; Love et al., 2014). Functional analysis of genes was based on KEGG (http://www.kegg.jp/ or http://www.genome.jp/kegg/), and homology markers were detected using Clustering Diagram v3.10.1 (Wu et al., 2015). Weighted correlation network analysis (WGCNA) is used to identify gene modules that exhibit coordinated expression and explore gene networks associated with the phenotypes of interest, with the aim of uncovering key genes within these network relationships. We used the package WGCNA v1.71 in R v4.1.0 for weighted gene coexpression network analysis (Langfelder and Horvath, 2008).

Based on the honeybee reference genome, event-level differential splicing analysis was conducted using rMATS version 4.0.2 (turbo). To quantify AS events, a ratio metric known as percent-spliced-in (PSI) value based on exons was employed. Normalization was performed using an efficient length of l. The PSI value was calculated for several classes of AS events, including skipped exons (SE), alternative 5′ splice sites (A5SS), alternative 3′ splice sites (A3SS), mutually exclusive exons (MXE), and retained introns (RI). Events with a *p* value less than 0.05 were deemed to have differential splicing between the A-BEE group and T-BEE group. In order to find similarities between the genes that are expressed or spliced differently in the honeybee brain and those in human with ASD, we aligned 3,531 high-quality reference protein sequences, which represent 948 known autism risk genes (SFARI and SPARK) were aligned against protein sequences associated with the honeybee genome using BLASTP with a two-way best matching strategy (Altschul et al., 1990; Zhang et al., 2022b). In total, 649 autism-associated protein sequences were matched with a similarity greater than 30% and an E value less than 0.000394.

SFARI: https://gene.sfari.org/.

SPARK for autism: http://spark-sf.s3.amazonaws.com/SPARK_gene_list.pdf.

### 2.9 Metabolomic analysis of honeybee intestine

Untargeted metabolomics was performed on six biological replicates from each group. Novogene Co., Ltd. (located in Beijing, China) conducted liquid chromatography-mass spectrometry (LC–MS) analyses. The analyses were conducted using a Vanquish Ultra-High-Performance Liquid Chromatography (UHPLC) system (Thermo Fisher Scientific) along with an Orbitrap Q Exactive HF-X Mass Spectrometer (Thermo Fisher Scientific), operating in both positive and negative polarity modes. Compound Discoverer 3.1 (Thermo Fisher Scientific) was used to process the raw data files produced by UHPLC–MS/MS to obtain accurate qualitative and relative quantitative results. The analysis of metabolomics data was conducted utilizing the MetaboAnalyst interface (version 5.0) software (Pang et al., 2021).

### 2.10 Statistical methods

#### 2.10.1 Metagenomic data

Microbial alpha diversity was evaluated according to richness and diversity (Richness/Shannon index) at the species level. Principal coordinate analysis (PCoA) based on the unweighted UniFrac distance matrices was used to evaluate the bacterial similarities between the A-BEE and T-BEE groups, and significance was determined using permutational multivariate analysis of variance (PERMANOVA). The gut bacterial abundances were compared between the A-BEE and T-BEE groups using the pairwise Mann–Whitney U test, with *p* values adjusted using the Benjamini-Hochberg procedure to decrease the false discovery rate (FDR).

#### 2.10.2 Transcriptomic data

Non-normally distributed continuous data were compared using the Mann–Whitney U test. The data were expressed as mean ± standard deviation or median (interquartile range). Data analysis was performed using GraphPad Prism v9.0.0 (86; Mitteer and Greer, 2022).

#### 2.10.3 Metabolomics data

Statistical analysis of the targeted metabolomics data was performed using MetaboAnalyst (version 5.0). The concentrations of the metabolites were not normalized, transformed, or scaled-in prior to statistical analysis. Features with >50% missing values were removed from the subsequent analysis. Statistical significance was determined using the Mann–Whitney U test with a *p* value < 0.05, and a marginal difference was defined as *p* < 0.05 and a fold change (FC) >2 (i.e., log2FC > 1).

#### 2.10.4 Multiomics association

We performed a correlation analysis of the normalized metagenome, transcriptome, and metabolome data. Pearson correlation was used to analyze the six biologically parallel means of each group (*p* < 0.05). Neural network graphs were visualized using Cytoscape v3.9.1 (Shannon et al., 2003).

## 3 Results

### 3.1 Basic information of enrolled children

**Figure 2:**
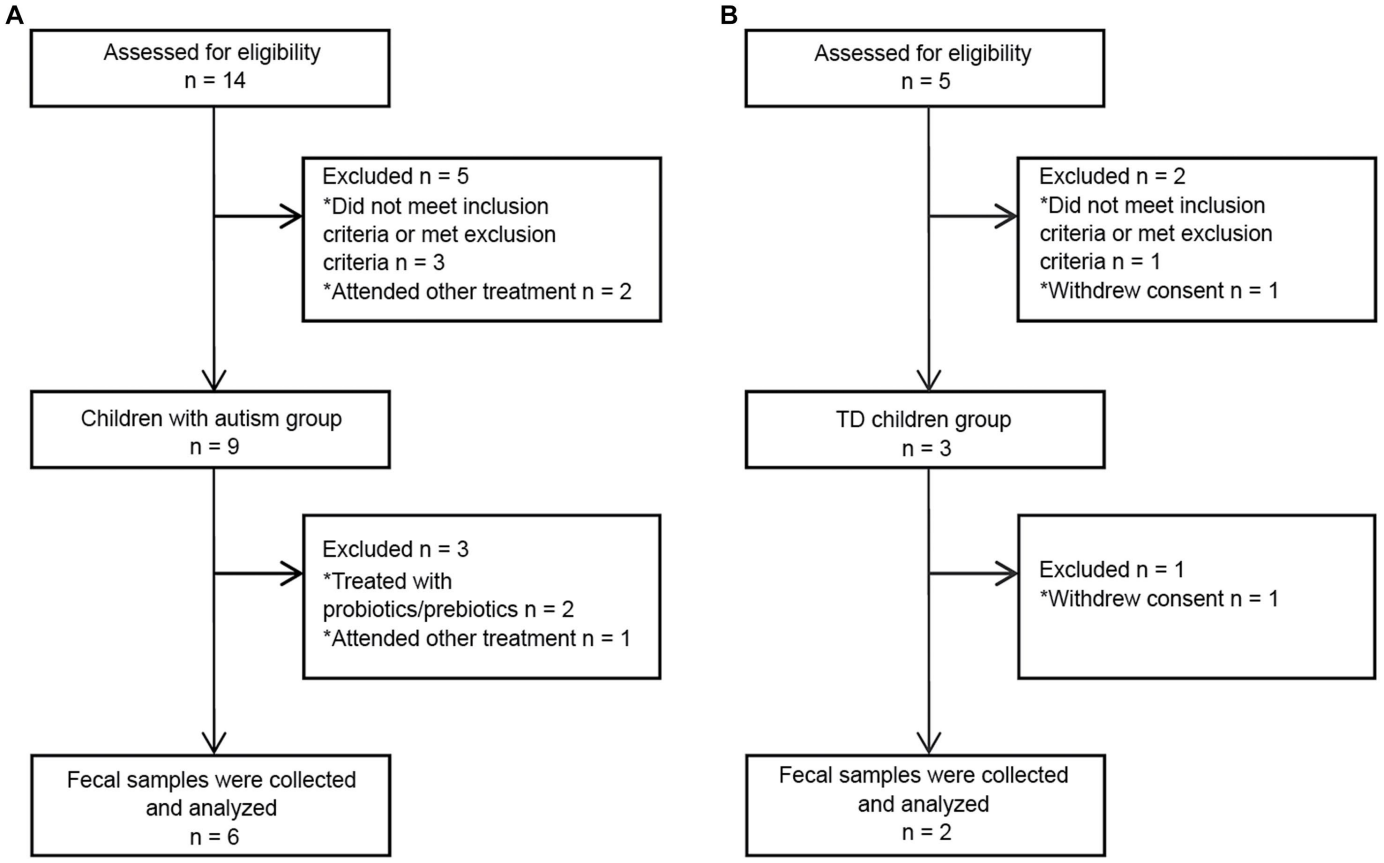
Flowchart of fecal donors in each group throughout the study. **(A)** A flowchart of the ASD-CHILD group. **(B)** A flowchart of the TD-CHILD group.

A total of 5 TD children were recruited during the study period. Of them, three were eventually excluded: one child had a family history of mental illness, and the guardians of two children withdrew midway through the study (Figure 2B). Therefore, two TD children participated throughout the entire study. Fourteen children with autism were admitted to Chinese PLA General Hospital outpatient clinic during the study period. Three children with autism were eventually excluded based on inclusion and exclusion criteria. Of the excluded children, one had a diagnosis of karyotype abnormalities, two had severe dietary preferences. The guardians of five children withdrew consent midway through the study, because they wanted their children to receive treatments beyond those permitted in the study (Figure 2A). According to CARS-2, all six children aged 3 to 9 years with severe autism were enrolled (Figure 1C; Supplementary Table S1). These items of CARS-2 were counted and plotted (Figure 1B). According to the score, the ASD-CHILD group exhibited imperfections in their relationships with people, imitation, and verbal communication, but they exhibited lower fear and nervousness scores. According to the clinical data, no child with autism was taking psychiatric medications in the study (Supplementary Table S1). And all the enrolled children had received behavioral treatment. The behavior therapy conducted by special institutions agreed by the government for ASD was mainly applied behavior analysis therapy (Granpeesheh et al., 2009). The children participating in the study did not experience severe dietary bias, and their nutritional status were good. The average body mass index (kg/m^2^) of the TD-CHILD group was 15.48, versus 15.73 in the ASD-CHILD group (Supplementary Table S1). The gastrointestinal symptoms of children with autism were evaluated using the GSI, and the results showed that the scores ranged from 3 to 6 (Figure 1C; Supplementary Table S1). The GSI scores reflected gastrointestinal symptoms in enrolled children with autism.

### 3.2 Fecal microbiota from children with autism impacts the learning and memory of microbiota-free honeybees

The A-BEE group exhibited differences in learning and memory abilities compared to the T-BEE group. After several rounds of training, more than half of the honeybees in the T-BEE group (i.e., TD-BEE 1 and TD-BEE 2) learned to recognize the conditioned stimulus (CS), whereas less than half of the honeybees in the A-BEE group (i.e., ASD-BEE 1–6) learned to recognize the CS (Figures 1A,D,E). After five rounds of training, the average correct response rates of the T-BEE and A-BEE groups were significantly different at 70.87 and 38.75%, respectively (*p* < 0.05, chi-square test). In the memory test, the memory accuracy of the TD-BEE 1 and TD-BEE 2 groups was 61.54 and 52.50%, respectively, whereas the memory accuracy of the ASD-BEE 1–6 groups was 12.50, 17.50, 15.00, 20.00, 25.00, and 35.00%, respectively (Figure 1F). Moreover, the average accuracy of the A-BEE group (20.83%) in the 2 h memory test was significantly lower than that of the T-BEE group (56.96%; *p* < 0.05, chi-square test; Figure 1G). In brief, the bees’ behavior was affected by the fecal microbiota in children with autism, which showed a decline in learning and memory abilities.

### 3.3 Transplanted bees can partially reproduce the intestinal microbiome of children with autism and TD children

**Figure 3:**
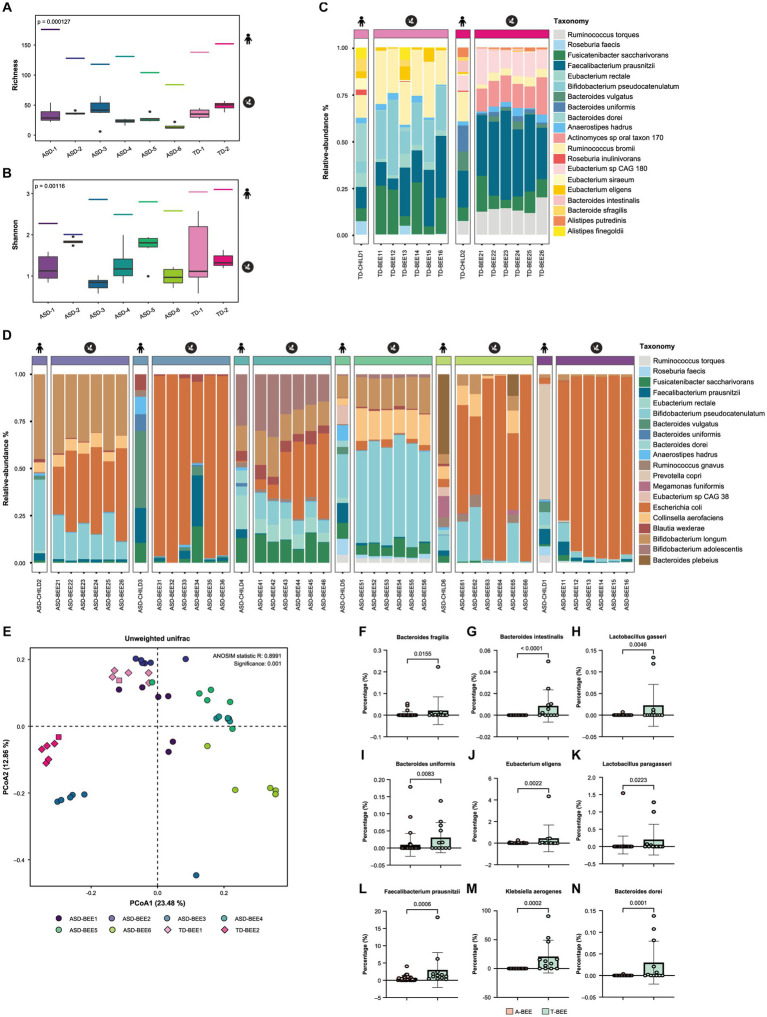
Colonization of honeybees with donor fecal microbiota. **(A,B)** The alpha diversity of the gut microbiota in honeybees was measured as a richness index, and the Shannon index from shotgun metagenomic sequencing revealed a significant difference between the A-BEE and T-BEE groups. The short line at the top of the figure indicates the alpha diversity of the donor fecal microbiota. Differences between groups were determined using the two-sided Mann–Whitney U test. **(C)** Percentages of the top 20 species in terms of abundance in honeybee gut microbiota in the T-BEE group (TD-BEE 1 and TD-BEE 2) and in the TD-CHILD group (TD-CHILD 1 and TD-CHILD 2). **(D)** Percentages of the top 20 species in terms of abundance in honeybee gut microbiota in the A-BEE group (ASD-BEE 1–6) and the abundance of the species in the ASD-CHILD groups (ASD-CHILD 1–6). **(E)** PCoA of unweighted UniFrac distances at the species level from the honeybee gut microbiota profiles of the A-BEE group (includes ASD-BEE 1–6) and the T-BEE group (includes TD-BEE 1 and 2). Differences between the groups were evaluated using PERMANOVA. **(F–N)** Normalized abundance of the species in the intestines of honeybees in the A-BEE and T-BEE groups. Differences between groups were determined using the two-sided Mann–Whitney U test.

Metagenomic sequencing and bioinformatics analyses of TD-CHILD, ASD-CHILD, T-BEE, and A-BEE samples revealed that the alpha diversity of the intestinal contents of colonized honeybees was lower than that of their child donor counterparts. Nevertheless, the between-group differences in the alpha diversity of the humanized honeybee intestinal contents were largely consistent with their corresponding donor counterparts (Figures 3A,B). At the species level, the percentages of the top 20 species in terms of abundance in the bee guts of the T-BEE and A-BEE groups were different. Although there were differences in species abundance between the intestinal microbiomes of honeybees colonized by donor fecal microbiota and the corresponding donor sample microbiomes, the composition ratio of the top 20 most abundant species was similar among the microbiomes (Figures 3C,D). In addition, beta diversity analysis results revealed consistency within the groups, whereas differences were evident between the T-BEE and A-BEE groups (Figure 3E). In other words, microbiota-free bee colonization of donor gut microbes can reproduce the structure and composition of donor gut microbes to some extent. To analyze bacterial colonization, a phylum level analysis was conducted. There was no significant consistent difference in the relative abundance of Bacteroidetes between children with autism and TD children. The relative abundance of Firmicutes in the intestinal tracts of children with autism was lower than that of TD children (Supplementary Figure S1B). The differences were analyzed at the species level, and the resulting volcano plot indicated 63 different strains between groups, of which 30 and 33 were enriched in the A-BEE and T-BEE groups, respectively (Supplementary Figure S1A). Although Bacteroidetes did not show significant consistent differences at the phylum level, there were more different bacteria at the species level, such as *Bacteroides fragilis* (*B. fragilis*), *Bacteroides intestinalis* (*B. intestinalis*), *Bacteroides uniformis* (*B. uniformis*), and *Bacteroides dorei* (*B. dorei*) which were significantly enriched in the T-BEE group but showed low relative abundance in the A-BEE group. Moreover, the relative abundance of *Eubacterium eligens* (*E. eligens*), *Faecalibacterium prausnitzii* (*F. prausnitzii*), *Klebsiella aerogenes* (*K. aerogenes*), *Lactobacillus gasseri* (*L. gasseri*), and *Lactobacillus paragasseri* (*L. paragasseri*) were significantly higher than in the A-BEE group’s bee guts (Figures 3F–N).

To evaluate the effect of fecal microbiota from ASD-CHILD donors on honeybee intestines, the length of their intestines was measured, including the midgut, ileum, and rectum. There was no significant difference in the length of the intestines among the groups (Supplementary Figures S1C,D). Hematoxylin–eosin staining was also performed on the ileum (Supplementary Figure S1E) and midgut (Supplementary Figure S1F) to identify any changes in the intestinal structure; however, no significant structural disparities in the intestine were observed among the experimental groups.

### 3.4 Fecal microbiota from children with autism impacts honeybee brain function by regulating gene expression and alternative splicing

**Figure 4:**
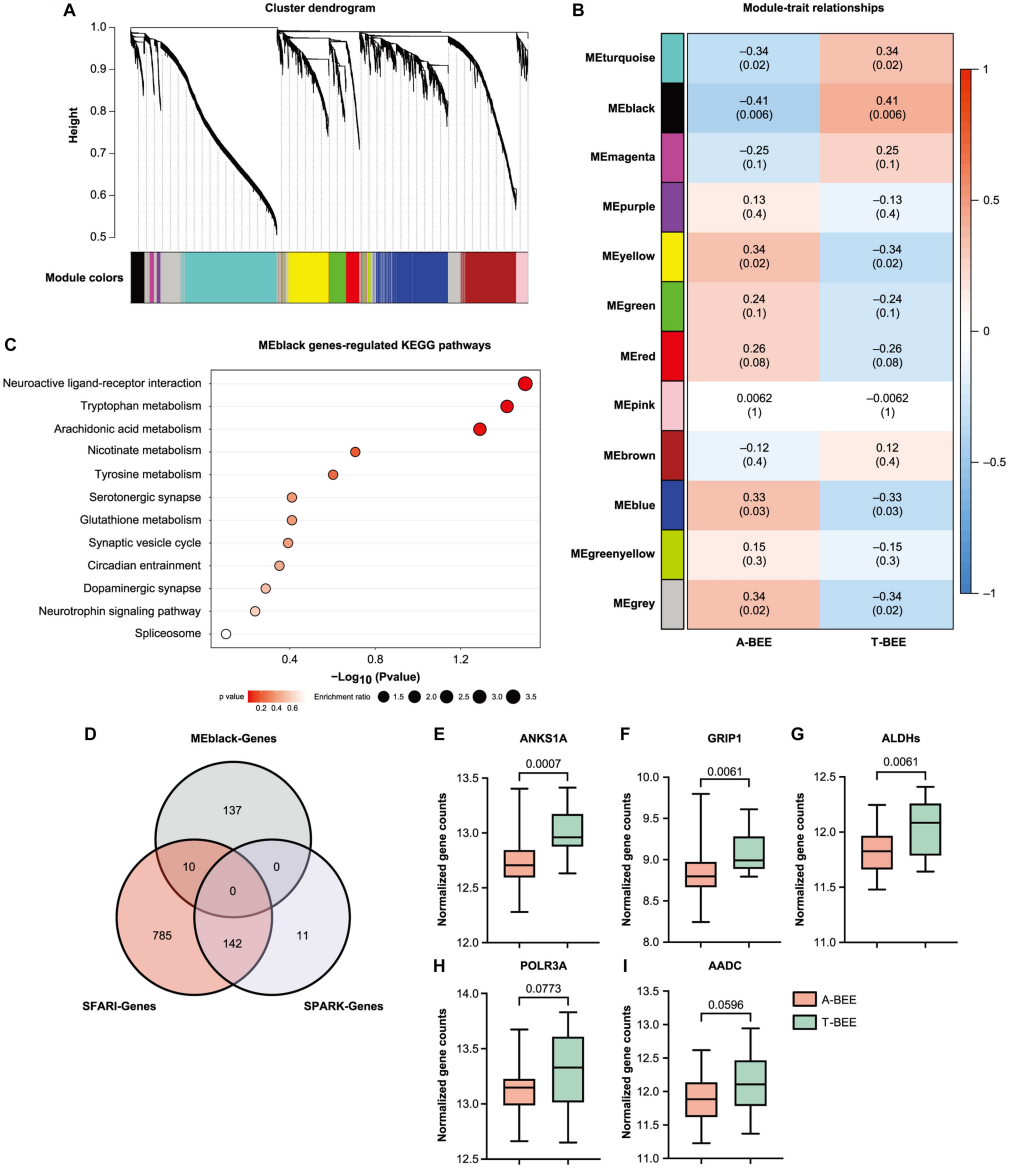
Fecal microbiota from ASD donors affects the expression of learning and memory-related genes in the brains of honeybees. **(A,B)** WGCNA revealed 12 gene modules (ME) that were correlated with different honeybee groups (the A-BEE group and the T-BEE group). Colored names represent gene modules assigned by the WGCNA pipeline. Heatmap colors indicate positive/negative Spearman correlation coefficients. The correlation coefficients are located above, and the *p* values are shown in parentheses below, within the squares. **(C)** KEGG enrichment pathways of MEblack genes (Fisher exact test). **(D)** Venn diagram of MEblack module genes and the overlap of these genes with the SPARK and SFARI gene data sets. **(E–I)** Normalized count of the genes in the brains of A-BEE and T-BEE honeybees. Differences between the groups were evaluated using the unpaired t test. ANKS1A: ankyrin repeat and SAM domain-containing protein 1A; GRIP1: glutamate receptor-interacting protein 1; ALDH: aldehyde dehydrogenase, mitochondrial; POLR3A: DNA-directed RNA polymerase III subunit RPC1; and AADC: aromatic-l-amino-acid decarboxylase.

The behavior of bees is associated with the expression of genes in their brains (Whitfield et al., 2003; Iino et al., 2023); therefore, transcriptomic sequencing was executed. To associate clusters of genes correlated with the A-BEE and T-BEE groups, WGCNA was performed. Honeybees inoculated with different intestinal microbes were used as the sample traits. Based on the gene expression in the honeybee brains, 12 modules (ME) were clustered using WGCNA (Figures 4A,B). The black module gene cluster (MEblack) was significantly correlated with different ASD-CHILD and TD-CHILD fecal microbiota-colonized honeybees (*p* < 0.001; Figure 4B). To determine between-group differences in the MEblack module of genes, a Wilcoxon rank-sum test was performed, which indicated that the expression of MEblack genes differed significantly between the A-BEE and T-BEE groups, i.e., it was downregulated in the A-BEE group (*p* = 0.023; Supplementary Figure S2). These genes were significantly enriched in synaptic signaling pathways, such as the serotonergic synapse, dopaminergic synapse, and synaptic vesicle cycle, as well as in amino acid metabolism pathways, such as tryptophan metabolism, nicotinate and nicotinamide metabolism, and glutathione metabolism (Figure 3C). We also compared these differentially expressed genes with the established brain-related gene sets of children with autism. Of the 147 genes in the MEblack module, 10 genes were found to intersect with the SFARI gene set (Figure 4D). Of the intersected genes, the differentially expressed genes ANKS1A (ankyrin repeat and SAM domain-containing protein 1A), GRIP1 (glutamate receptor-interacting protein 1), ALDHs (aldehyde dehydrogenase, mitochondrial), POLR3A (DNA-directed RNA polymerase III subunit RPC1) and AADC (aromatic-l-amino-acid decarboxylase) were associated with basal brain development, blood–brain barrier function, substance metabolism, and neural synapse functions (Figures 4E–I; Wassenberg et al., 2017; Harting et al., 2020; Tan et al., 2020; Ryu et al., 2021).

**Figure 5:**
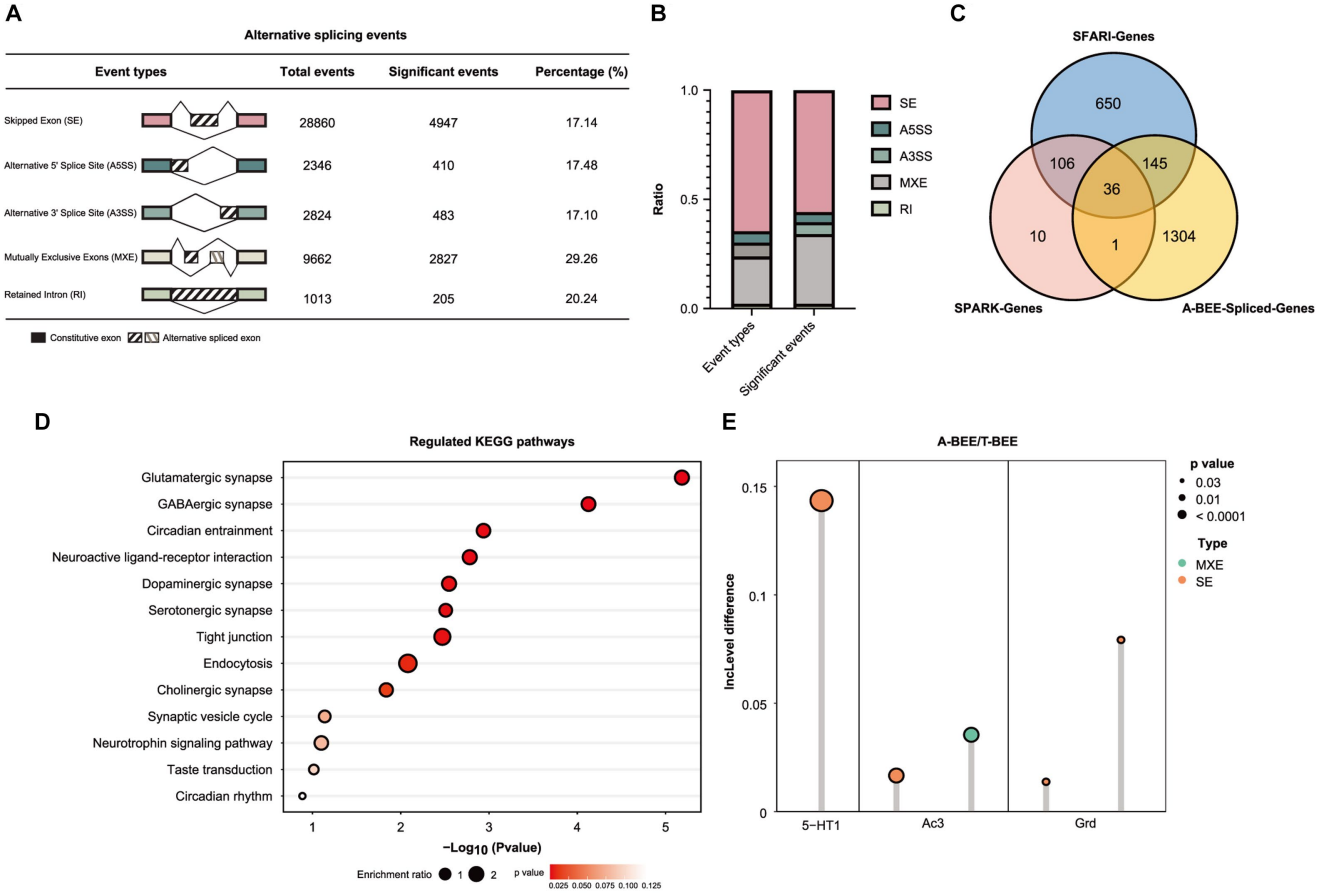
Fecal microbiota of children with autism affect AS in honeybee brains. **(A)** Number of the differential AS events in the brains of honeybees in the A-BEE group compared with the T-BEE group. **(B)** Stacked column graph showing the relative abundance of different types of AS events. A3SS: alternative 3′ splice site; A5SS: alternative 5′ splice site; MXE: mutually exclusive exon; RI: retained introns; SE: skipped exon. **(C)** Venn diagram of differentially spliced genes in the brains of honeybees in the A-BEE and T-BEE groups (gnotobiotic bee spliced genes; FDR <0.05), and their overlap with SPARK and SFARI gene datasets. **(D)** KEGG pathways regulated in the brains of the A-BEE group honeybees based on differentially spliced genes (Fisher exact test). **(E)** Differentially spliced events in the 5-HT1, AC3, and GRD gene present in both the SPARK and SFARI gene data sets. 5-HT1: serotonin receptor; AC3: adenylate cyclase 3; and GRD: GABA-gated ion channel.

Moreover, the AS of bee brains’ genes was also correlated with fecal microbes. KEGG functional enrichment analysis of the differentially expressed genes in the honeybee brain (MEblack) suggested the involvement of the spliceosome pathway (Figure 4C). Therefore, different AS events in the brains of honeybees colonized with fecal microbes from ASD-CHILD and TD-CHILD donors were analyzed. According to rMATS analysis of the different native splicing events of brain-related genes, 44,705 events were detected, with SE being the most abundant among the different AS types. Events accounting for approximately 20% of each AS type exhibited significantly different inclusion rates among the A-BEE groups, with multiple exclusive exceptions exhibiting the highest proportion of significant events (Figures 5A,B). In total, 1,486 genes were involved in differential AS events, of which 181 intersected with the SFARI gene set and 37 intersected with the SPARK gene set (182 genes in total). Interestingly, almost all homologous genes identified belong to the SFARI gene set that was associated with ASD with high confidence (Figure 5C). Enrichment analysis of the differentially spliced genes revealed that the enriched KEGG pathways regulated in the brains of the A-BEE and T-BEE groups included glutamatergic synapses, serotonergic synapses, GABAergic synapses, and dopaminergic pathways (Figure 5D). The enrichment analysis results also revealed that some genes, such as 5-HT1 (serotonin receptor), AC3 (adenylate cyclase 3), and GRD (GABA-gated ion channel), were involved in multiple pathways related to neural synapses, and these genes were differentially expressed in the A-BEE and T-BEE groups. Furthermore, different AS events could occur in a single gene and exhibit different inclusion rates (Figure 5E). Thus, fecal microbiota from ASD-CHILD donors can induce differential gene expression in the brains of honeybees while also inducing AS, thereby affecting genes that are essential for the social behavior of bees and are also associated with ASD.

### 3.5 Fecal microbiota from children with autism impacts intestinal metabolites in honeybees

**Figure 6:**
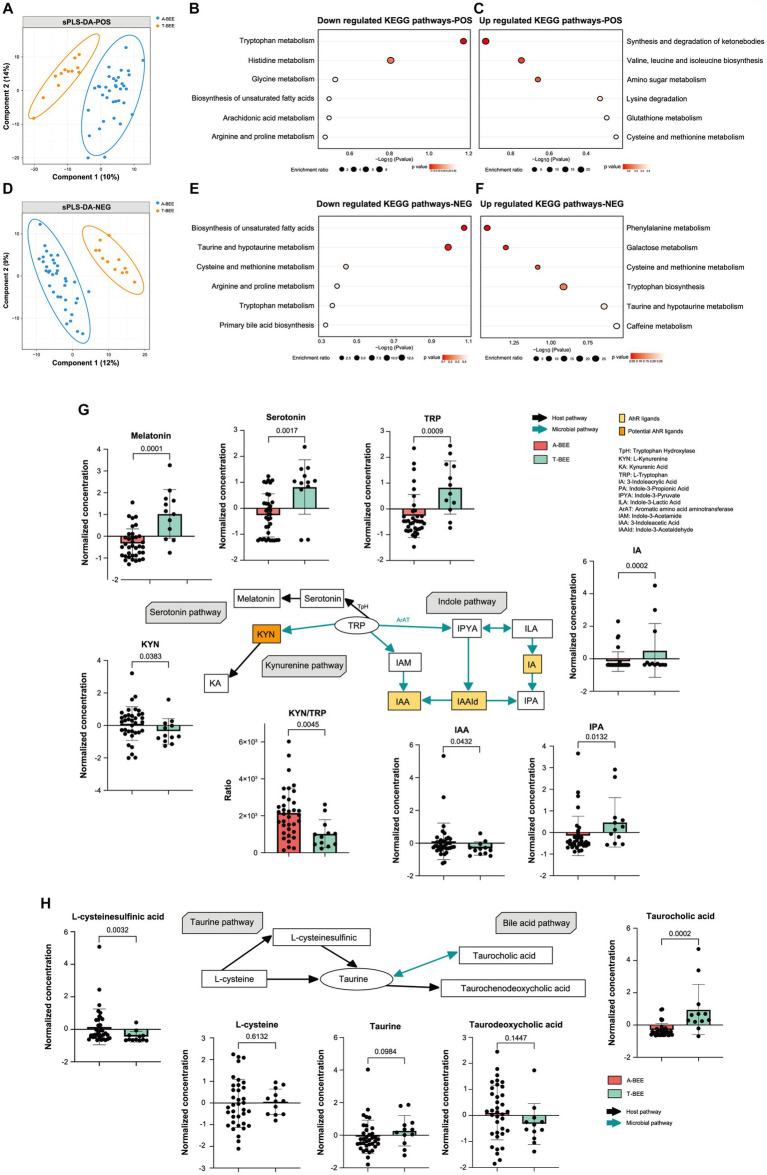
Colonization of honeybees with the fecal microbiota of children with autism affects intestinal metabolism. **(A)** Sparse partial least squares discriminant analysis based on all metabolites detected in the positive polarity mode in the gut of honeybees. Sample plots were clustered with 95% confidence intervals. **(B,C)** KEGG enrichment pathways downregulated and upregulated in the hemolymph of honeybees in the A-BEE group compared with the T-BEE group based on differentially regulated metabolites (Fisher exact test). **(D)** Sparse partial least squares discriminant analysis based on all metabolites detected in the negative polarity mode in honeybee guts. Sample plots were clustered with 95% confidenceintervals. **(E,F)** KEGG enrichment pathways downregulated and upregulated in the hemolymph of honeybees in the A-BEE group compared with the T-BEE group based on differentially regulated metabolites (Fisher exact test). **(G)** Tryptophan metabolism via the kynurenine, serotonin, and indole pathways. Green arrow: involvement of gut bacteria in the process. Black arrow: primarily host-mediated process. Box plot shows the differences in the differential metabolites in the three pathways between the A-BEE and the T-BEE groups. Group differences were tested using the two-sided Mann–Whitney U test. **(H)** Metabolites involved in the formation of taurine via the taurine synthesis and bile acid metabolism pathways. Box plot shows the differences of the differential metabolites in the two pathways between the A-BEE and the T-BEE groups. Group differences were evaluated using the two-sided Mann–Whitney U test.

Nontargeted metabolomics and gas chromatography/mass spectrometry were used to analyze the intestinal contents of honeybees. The metabolic profiles of the A-BEE group and T-BEE group honeybees were significantly different in both positive and negative polarity modes (Figures 6A,D). To determine the differential metabolites between the groups, DESeq2 was used to conduct a difference analysis on the data obtained from the detection of negative and positive polarity modes. Then, according to KEGG enrichment analysis of the differentially upregulated and downregulated metabolites in the T-BEE and A-BEE groups (Figures 6B,C,E,F), the upregulated and downregulated pathways in the A-BEE group included the tryptophan, taurine, and bile acid metabolism pathways; therefore, the altered substances involved in these metabolism pathways were explored. A two-sided Mann–Whitney U test was used to determine the differences between the groups in terms of the substances in these pathways (Figure 6G). The differential metabolites were largely involved in the tryptophan metabolism pathway. Dietary tryptophan can be broken down by intestinal microbes into various indole derivatives, such as 3-indole acrylic acid (IA) and indole-3-pyruvate (IPA), which are one of key components of homeostasis in the intestine (Agus et al., 2018). Additionally, tryptophan can be broken down by the host into serotonin and melatonin, which are substances associated with cognitive ability. It is also metabolized by the kynurenine pathway, which is mediated by host enzymes. It could affect the production of kynurenine and kynurenic acid. Compared with the T-BEE group, melatonin and serotonin in the A-BEE group were significantly downregulated in the serotonin metabolism pathway; IA and IPA were also downregulated in the indole pathway, and L-kynurenine was enriched in the kynurenine pathway. Furthermore, the L-kynurenine/L-tryptophan ratio was significantly higher in the A-BEE group than in the T-BEE group (*p* < 0.05). In conclusion, the colonized microbiome in the honeybee intestine was involved in tryptophan metabolism, possibly by regulating the conversion of tryptophan to indole derivatives, serotonin, and kynurenine.

The honeybee intestinal microbiome also regulated the metabolism pathway of taurine, a metabolite involved in nervous system function. Taurine can be generated via the taurine synthetic pathway or from the bile acid metabolism pathway. Both L-cysteine and L-cysteine sulfinic acid are precursors of taurine in the taurine synthesis pathway. L-cysteine levels did not differ among the groups, whereas L-cysteine sulfinic acid was upregulated in the A-BEE group (Figure 6H). The bile acid metabolism pathway, which involves microorganisms, was also analyzed, and taurocholic acid was significantly enriched in the T-BEE group. Taurocholic acid can be hydrolyzed into taurine under the joint mediation of microorganisms and hosts. This partly explains why A-BEE group honeybees had more precursors for taurine synthesis in their intestine but not more taurine than T-BEE group honeybees, i.e., more taurine was produced in the intestinal tract of the T-BEE group honeybees through the bile acid metabolism pathway than in the intestinal tract of A-BEE group honeybees via this pathway.

### 3.6 Association analysis of differential bacteria and gut metabolites in the honeybee gut and differently expressed genes in the honeybee brain

**Figure 7:**
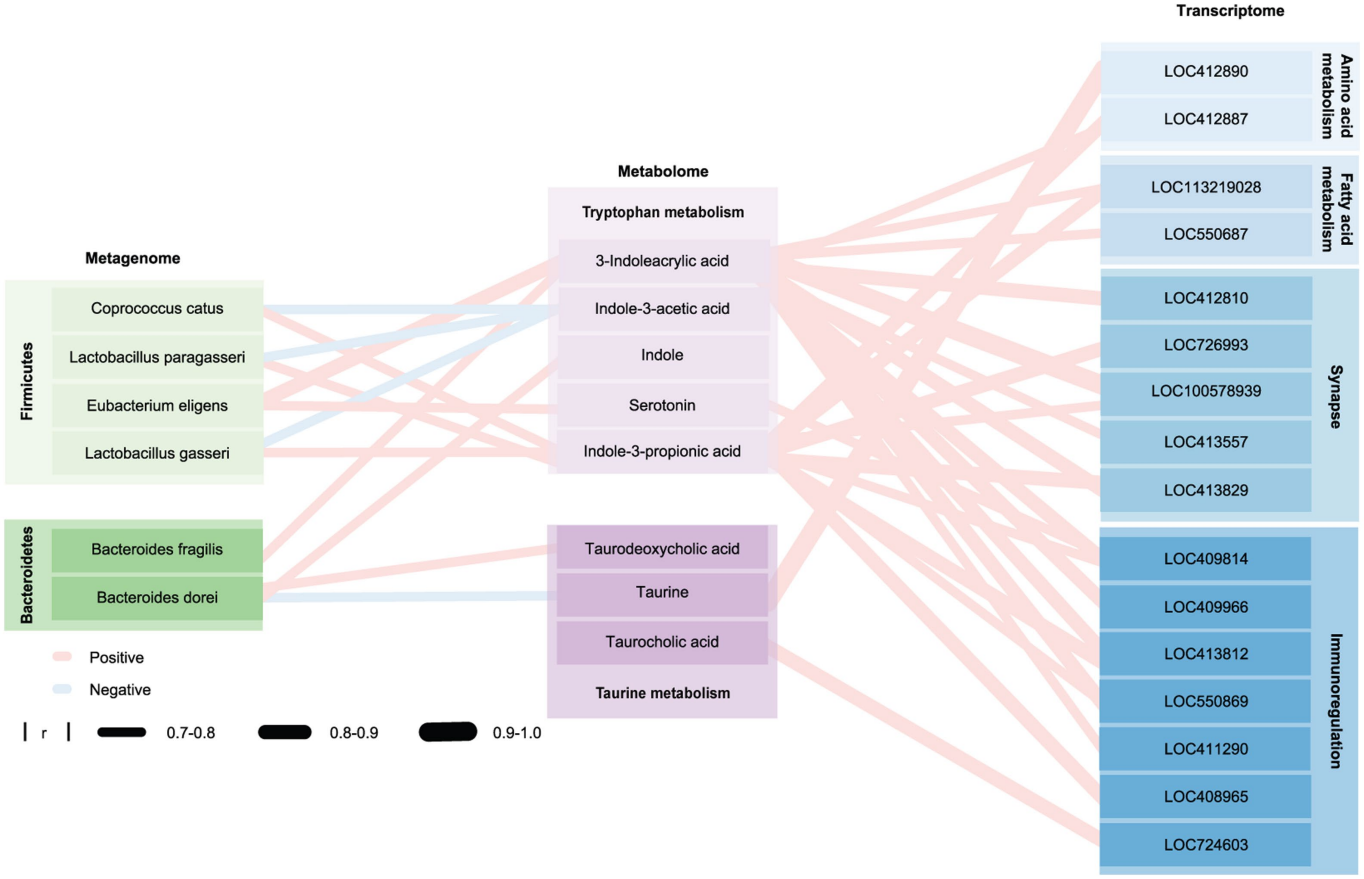
Honeybee gut microbiota affect the honeybee brain through metabolites. Network diagram of the macrogenome, transcriptome, and metabolome drawn using Cytoscape. Blue and red edges indicate positive and negative correlations, respectively; edge width represents the correlation strength. Nodes shown in different shades of green, purple, and blue represent different phyla, metabolites in different metabolic pathways, and genes involved in different pathways, respectively. Group differences were determined using a Spearman rank test.

Compared with honeybees colonized by TD-CHILD fecal microbiota, honeybees colonized with fecal microbiota from ASD-CHILD donors exhibited different microbiota structures and intestinal metabolomics as well as different brain-related gene expression and impaired cognitive abilities. To explore the relationship between these groups, an association analysis using Pearson’s correlation was conducted. First, association analysis using the metagenomics and metabolomics data was performed and a correlation analysis thermogram was plotted by performing association analysis of the different strains and substances involved in the pathway of interest (Supplementary Figure S3A). Subsequently, association analysis using metabolomics and transcriptomics data was performed, and a correlation analysis heat map was plotted according to the association analysis between substances involved in the pathway of interest and genes in the MEblack module (Supplementary Figure S3B). The results showed that some strains deficient in the A-BEE group were significantly positively correlated with the metabolites of the tryptophan-indole metabolism and bile acid hydrolysis pathways, both of which involve the participation of intestinal bacteria, and these metabolites were significantly positively correlated with some genes in the MEblack module (|r| > 0.7, Figure 7). Interestingly, most identified strains belonged to the phylum Firmicutes and the phylum Bacteroidetes, such as *E. eligens*, *L. gasseri*, *B. fragilis*, and *B. dorei*. Genes were related to synaptic function according to functional annotation. For example, both *E. eligens* and *B. fragilis* exhibited positive correlations with 3-IA, and this metabolite was also positively correlated with the expression of genes that regulate synaptic function, including LOC100578939 (single Ig IL-1-related receptor, SIGIRR). Furthermore, *B. dorei* belongs to the phylum Bacteroidetes, which is related to taurodeoxycholic acid and taurine in the taurine pathway. These metabolites were strongly associated with LOC412890 (A disintegrin and metalloproteinase with thrombospondin motifs 3, ADAMTS3), which is involved in regulating amino acid metabolism in bee brains.

## 4 Discussion

Several studies have confirmed that many neuropsychiatric diseases are closely related to intestinal microecology (Petra et al., 2015; Quigley, 2017; Generoso et al., 2021). Children with autism mainly exhibit psychoneurological symptoms, and gastrointestinal dysfunction and immune imbalance are prevalent in these studies, suggesting that the microbiome is involved in ASD (Adams et al., 2011; Lai et al., 2014; Petra et al., 2015; Madra et al., 2020). Numerous studies have investigated the microbiomes and metabolomes of children with autism compared with TD control groups (Coretti et al., 2018; Kang et al., 2018; Jones et al., 2022). In addition, animal experiments have confirmed that treatment with fecal microbiota from children with autism promotes behavior in mice associated with the core behavioral characteristics of ASD (Sharon et al., 2019). In the present study, we used honeybees as a model for the first time to investigate the effects of fecal microbiota from children with autism on host pathophysiology and behavior. We found reduced cognitive performance in A-BEE group honeybees compared to T-BEE group honeybees, which were colonized with ASD-CHILD and TD-CHILD fecal microbiomes, respectively. Notably, our findings demonstrate that alterations in the gut microbiota of children with autism can influence the host’s nervous system and cognitive behavior without establishing a causal relationship between gut microbiota and ASD.

The gut microbiota is one of the crucial factors in gut-brain communication. Through metagenomic analysis, we hope to identify potential bacterial candidates that may contribute to the observed behavioral differences in honeybees. Compared with the T-BEE group, some species were lacking or low in abundance in the A-BEE group, e.g., *B. fragilis*, *B. intestinalis*, *B. uniformis*, *E. eligens*, *F. prausnitzii*, *Klebsiella aerogenes*, *L. gasseri*, *L. paragasseri*, and *B. dorei*. In a cohort study from China, the abundance of *B. fragilis* was lower in children with autism than in TD children and was accompanied by the downregulation of the tryptophan metabolism pathway (Zou et al., 2020). Furthermore, research suggests that oral administration of the human commensal *B. fragilis* to ASD mice modulated autism-related intestinal physiology and improved behavioral abnormalities (Hsiao et al., 2013). *B. uniformis* was found to impact the reward response in the rat brain of food-addicted rats and reduce their anxiety (Agustí et al., 2021). Interestingly, two randomized, controlled, double-blind placebo trials found that supplementation with *L. gasseri* relieves chronic stress, a finding that has been validated in multiple animal studies (Kang et al., 2010; Kim et al., 2018; Nishida et al., 2019). Bacteria including *B. intestinalis*, *E. eligens*, *F. prausnitzii*, and *B. dorei*, are involved in the regulation of host immune function and play important roles in maintaining host immune balance (Chung et al., 2017; Lopez-Siles et al., 2017; de Filippis et al., 2021; Pereira et al., 2021; Yasuma et al., 2021). Interestingly, *K. aerogenes*, the first recorded human intestinal symbiont sensitive to pineal gland/gastrointestinal melatonin, was enriched in the T-BEE group. This species is considered the biological clock of the intestinal microbiome because it can sense the secretion of melatonin and simultaneously express circadian rhythms (Paulose et al., 2019; Graniczkowska et al., 2022). These results partly explain the behavioral changes observed in honeybees colonized by the intestinal microbiome of ASD-CHILD donors. Although definitive causality between these differential gut bacteria and behavioral changes in hosts cannot be established, the experimental evidence suggests that these gut bacteria hold potential research value on the correlation between ASD and gut microbiota.

Intestinal microbiota impact gene expression and host behavior through various means, among which the regulation of intestinal metabolism allows intestinal microbiota to send regulatory signals to the host central nervous system (Rogers et al., 2016; Morais et al., 2021). We found that the intestinal microbiota of ASD-CHILD donors affected the intestinal metabolism of honeybees through multiple pathways, including the tryptophan and taurine metabolism pathways. Taurine is essential for brain development (Benrabh et al., 1995; Kilb and Fukuda, 2017; Oja and Saransaari, 2022). We found higher taurine levels in the intestines of T-BEE group honeybees than in the intestines of A-BEE group honeybees. Increased taurocholic acid levels were also found in the intestines of T-BEE group honeybees, which can be further hydrolyzed into taurine by intestinal microorganisms. Supplementation with royal jelly to upregulate taurine metabolism pathways has shown potential for ameliorating cognitive decline in aging bees (Chen et al., 2017). Furthermore, the modulation of bee brain sensitivity through taurine supplementation can enhance and consolidate nursing and foraging behaviors (Han et al., 2017). Interestingly, one study observed that mice colonized with ASD fecal microbiota also exhibited differences in the taurine metabolism pathway by studying mouse autism models (Golubeva et al., 2017). The differential metabolites between the T-BEE and A-BEE groups were more enriched in the tryptophan metabolism pathway. Compared with T-BEE group honeybees, higher L-kynurenine levels were found in the intestines of A-BEE group honeybees, whereas the levels of melatonin and indole pathway-related substances were reduced. Metabolic disorders of the kynurenine pathway are closely related to nervous system diseases (Zhang et al., 2020; Chen et al., 2021; Mithaiwala et al., 2021). The upregulation of the kynosurine pathway causes marked autistic-like behaviors, repetitive stereotyped behavior, worse learning and memory abilities, and a despairing mood in rats (Kong et al., 2022). As an immunomodulator, antioxidant, and neuroprotective agent, melatonin is inversely associated with brain inflammation and neurodegenerative diseases. Moreover, several studies have found a relationship between melatonin and ASD (Hardeland et al., 2006; Tordjman et al., 2017; Hardeland, 2019; Cardinali, 2021). Melatonin is involved in the neurogenesis and plasticity of neurogenic neural stem cells and in the development of the fetal brain (Jin et al., 2018; Lord et al., 2018; Wu et al., 2020). An fMRI study on the central nervous system found that melatonin can induce sleep-like changes, which in turn affect the language and memory processing functions of the hippocampus and improve memory (Gorfine et al., 2007). In studies pertaining to bees, it was found that melatonin significantly influenced their social behavior of bees across various age groups and was involved in regulating their division of labor (Yang et al., 2007). Furthermore, melatonin was reported to enhance the antioxidant capacity of bees and safeguard worker bees against pesticide effects during foraging activities (Li et al., 2022). In the present study, more IA and IPA were produced by the T-BEE group than by the A-BEE group in the indole pathway, in which IA has the AhR ligand. In our previous research, we found that higher levels of AhR ligands improve learning and memory abilities in honeybees, but the inhibition of AhR can impair these abilities (Zhang et al., 2022a). Overall, the gut microbiome influences brain function through multiple metabolic pathways, including more intricate and multifaceted mechanisms.

The behavior of honeybees is closely related to gene expression in their brains, and homologous molecular mechanisms underlying the social responses of honeybees and humans are well-documented (Shpigler et al., 2017; Zhang et al., 2022b). Compared with the T-BEE group, we found that a set of differentially expressed genes in the brains of A-BEE group honeybees were downregulated, e.g., those involved in receptor interaction, tryptophan metabolism, and serotonergic and dopaminergic synapse pathways, which are associated with critical brain functions. Synaptic plasticity is a key mechanism of higher brain functions, and one form of synaptic plasticity is related to the regulation of AMPAR abundance and properties. The gene LOC408965 encodes GRIP1. GRIP1 is an AMPAR (α-amino-3-hydroxy-5-methyl-4-isoxazolepropionic acid receptor) binding protein involved in the regulation of the traffic and synaptic targeting of AMPARs. The AMPAR level determines the synaptic excitation intensity, which is necessary for maintaining learning and memory functions (Trotman et al., 2014; Tan et al., 2020). Previous studies have found that mice exhibiting reduced expression of brain-encoded genes develop several neuroses, including reduced memory capacity (Wang G. et al., 2022). In addition, studies of individuals with autism have shown that the gene that encodes GRIP1 in the brain is downregulated, which is associated with neurological symptoms (Åhsgren et al., 2005; Mejias et al., 2011; Choudhury et al., 2012). Notably, ASD-related genes are not only disrupted in terms of their expression levels but also AS in the bee brain, consistent with previous study findings (Chang et al., 2022; Zhang et al., 2022a,b). The AS genes were found to overlap with the SFARI gene data set, which is associated with human autism and disturbed social responses in bees (Figure 6). We found more differentially AS genes in the brains of honeybees in the A-BEE group, which were present in both the SPARK and SFARI gene data sets, than did a mouse autism model study (Sharon et al., 2019). Correspondingly, KEGG enrichment analysis of differentially expressed AS genes that overlapped with this data set revealed that the genes were significantly enriched in glutamatergic synapse, serotonic synapse, GABAergic synapse, and dopaminergic pathways as well as neuroactive ligand-receptor interaction, taste transduction, and circadian rhythm pathways, which are related to synaptic function and widely involved in nervous system functions (Chattopadhyaya, 2011; Frenguelli, 2022). Genes in the enrichment pathway exhibited different inclusion rates, e.g., 5-HT1, which encodes a serotonin receptor known to be involved in higher brain functions and behavior (Lucki, 1991; Kosari-Nasab et al., 2019). In a previous study, 5-HT1B-knockout mice were more aggressive and reactive than wild-type mice, and 5-HT1A-knockout mice exhibited more anxiety than wild-type mice (Zhuang et al., 1999). The 5-HT1A agonist 8-hydroxy-2-(di-n-propyl amino) tetralin improves autism-related behaviors in the offspring of mice exposed to valproic acid (Wu et al., 2018). Taken together, the findings suggest an association between FMT and altered gene expression in the bee brain, which may account for the impaired learning and memory abilities observed in bees colonized with fecal microbiota from children with autism.

Intestinal metabolites act a bridge between the intestinal microbiome and the central nervous system (Massey et al., 2023; Wang T. et al., 2023). Therefore, we tried to explore the relationships among metabolites, intestinal bacteria, and brain gene expression. We found that substances in the tryptophan and taurine metabolism pathways are associated with more than one bacterial species and related to the expression of multiple brain genes (| r | >0.7, *p* < 0.05). And these bacteria mainly belong to Bacteroidetes and Firmicutes. Interestingly, in a study, it was also found that the guts of BTBR mice displayed impaired bacteria-mediated bile transformation and serotonin production, while at the phylum level, it displayed changes in the abundance of Bacteroidetes and Firmicutes (Golubeva et al., 2017). Bacteria belonging to the phylum Firmicutes, including *E. eligens*, *L. paragasseri*, and *L. gasseri*, and the phylum *Bacteroides*, including *B. dorei*, and *B. fragilis*, had a lower relative abundance in the A-BEE group was reduced and these bacteria were positively associated with several key metabolites of the tryptophan pathway. We found that both *E. eligens* and *B. fragilis* are positively correlated with 3-IA. Bee brain genes, such as SIGIRR, which have positively correlate with 3-IA, are involved in the synaptic function. According to the results, we speculated that *E. eligens* and *B. fragilis* affect the brain functions of the host through the regulation of the tryptophan-indole metabolism pathway. In addition, we found that *B. dorei*, which belongs to Bacteroidetes, is associated with taurodeoxycholic acid and taurine in taurine pathway. Although these findings have undergone rigorous scientific analysis, they solely reflect the potential impact of gut bacteria on the host rather than establishing a causal relationship. Further investigation is warranted to elucidate the molecular mechanisms underlying these multiple omics associations.

The composition and physiological functions of the human gut microbiota are highly complex, posing challenges for replication in animal models. Moreover, ASD, as a neurodevelopmental disorder primarily characterized by social impairment, further complicates research on animal models. Our study represents a pioneering effort by utilizing honeybees colonized with intestinal microorganisms from children with autism as a model to investigate the interplay between ASD and microbiome structure, intestinal metabolism, brain expression, and cognitive behavior. While this study is an innovative project that contributes valuable insights into ASD microbiota research, it has certain limitations. As mentioned before, honeybees are social animals, offering a unique perspective compared with other existing models. However, as a recently developed experimental model, bees have certain limitations. First, because global research using bees as experimental models is unusual, knowledge about these bee models is scarce and the body of literature and the number of comparative investigations are limited. Second, despite significant genetic similarities between the brain genomes of honeybees and humans and numerous shared genes, the evolutionary distance between insects like bees and mammals such as germ-free mice or rats remains substantial. Third, the bees used in the experiment are worker bees. All worker bees are female, which has potential bias in studying human diseases, especially those with gender differences. Moreover, the small body of bees implies that they have a short intestinal length and small intestinal capacity. Consequently, the bee intestine does not provide a strictly anaerobic environment similar to that of humans, making it a less favorable environment for the growth of strict anaerobes. Thus, some facultative anaerobes, such as *Escherichia coli* (*E. coli*) may have an advantage in bee gut colonization. As in other insects (Johnson and Barbehenn, 2000), the oxygen content in different parts of the bee intestine is not identical. Our study only collected the entire intestine of bees for gut microbiota analysis. These physiological characteristics may explain why the level of *E. coli* in bees that had undergone FMT was higher than expected despite having a greater variety of bacterial species than anticipated. Furthermore, there are differences in bee dietary habits compared to humans, which may affect the colonization of human gut microbiota in the guts of honeybees. However, different species have different dietary habits, and there are varying degrees of differences in dietary habits compared to humans, whether it is the widely used classic animal models such mice and rats, or other animal models that are more closely related to humans. This is a common problem faced by animal model research and one of the limitations of our study. Although bees have potential as models for studying human gut microbiota, they may not fully reflect all processes occurring in the human gut microbiota owing to their own characteristics. Additionally, our study included fecal samples from both ASD and healthy children who do not possess native bee microbiota, thereby posing a risk of tissue damage to bee intestines. Intestinal length serves as an important indicator reflecting intestinal inflammation of honeybee populations (Chang et al., 2022). Therefore, in our study, the influence of both intestinal tissue structure and length was considered to minimize any influence of intestinal damage on behavioral studies in our bee model. Notably, we did not find any significant differences in gut structure and length between honeybees colonized with fecal microbiota from children with autism and those colonized with fecal microbiota from TD children, suggesting a certain level of tolerance toward human fecal microbiota within honeybee intestines. In our study, all participants underwent meticulous screening based on predefined inclusion and exclusion criteria to ensure a certain degree of representativeness, whereas the small number of fecal donors is a limitation of our research. Therefore, a comprehensive comparative analysis of fecal microbiota was not conducted in the children or honeybees, and only an objective presentation was provided. Moreover, some studies have reported that the majority of ASD patients are male (Wang J. et al., 2022; Tofani et al., 2023). Considering the gender characteristics of experimental bees, gender was not considered in this study, and thus the ASD subjects included in this study not reflecting the known gender ratio in ASD. In addition, there are still some patients who have not found any accompanying gastrointestinal symptoms. Therefore, the subjects with autism included in this study had gastrointestinal symptoms, who may not be representative of the entire ASD population. The dietary status is a key factor affecting gut microbiota, and there are many indicators to evaluate it, such dietary preferences and dietary intakes (Seo and Je, 2018). We excluded the influence of dietary preferences in the study, but did not quantify dietary intake. A survey of dietary intakes can comprehensively evaluate the impact of dietary intakes on research. In this study, we did not conduct a quantitative survey of dietary intakes among enrolled children, and used BMI as an indirect evaluation indicator. Although the BMI can indirectly reflect dietary intake (Descarpentrie et al., 2023; Yoong et al., 2023), not including dietary intakes is one of the limitations in the study. Nevertheless, our findings support the hypothesis that gut microbiota in children with autism can influence host brain function and behavior to some extent. Furthermore, we present supplementary evidence supporting bees as a novel model for studying microbiota-gut-brain interactions in ASD. Our team intends to conduct a larger-scale investigation to delve deeper into the intriguing findings for mechanistic elucidation.

In conclusion, this pioneering study explores bees as a model for human gut microbiota research, revealing impactful insights into the relationship between gut microbiota and ASD. Our findings underscore the influence of autism-associated microbiota on bee cognition and metabolism, highlighting significant gene overlap with human ASD gene sets. Thus, further in-depth exploration of the mechanisms of the microbiota-gut-brain axis in ASD is necessary.

## Data availability statement

The datasets presented in this study can be found in online repositories. The names of the repository/repositories and accession number(s) can be found in the article/Supplementary material.

## Author contributions

YL: Methodology, Visualization, Writing – original draft, Writing– review & editing. YZ: Data curation, Validation, Visualization, Writing – review & editing. XL: Data curation, Investigation, Writing – review & editing. YM: Software, Writing – review & editing, Methodology. ZZ: Methodology, Writing – review & editing, Validation. HZ: Conceptualization, Project administration, Supervision, Writing – review & editing. YY: Conceptualization, Funding acquisition, Resources, Writing – review & editing.
