# Supplementary material for: The fecal microbiota from children with autism impact gut metabolism and learning and memory abilities of honeybees
Source: Front Microbiol. 2023 Nov 23;14:1278162. doi: 10.3389/fmicb.2023.1278162 (PMC10704100; doi:10.3389/fmicb.2023.1278162)
Supplement: Supplementary file 1 [file Data_Sheet_1.PDF]

Supplementary Material

The Fecal Microbiota from Children with Autism Impact Gut Metabolism and Learning and Memory Abilities of Honeybees

Yiyuan Li [1,2], Yan Zhang[1], Xi Luo[1,2], Yujie Meng[3], Zhaopeng Zhong[3], Hao Zheng[3]*, Yunsheng Yang[1]*

*** Correspondence:**

Yunsheng Yang: sunnyddc@plagh.org

Hao Zheng: hao.zheng@cau.edu.cn

Supplementary Figures and Tables

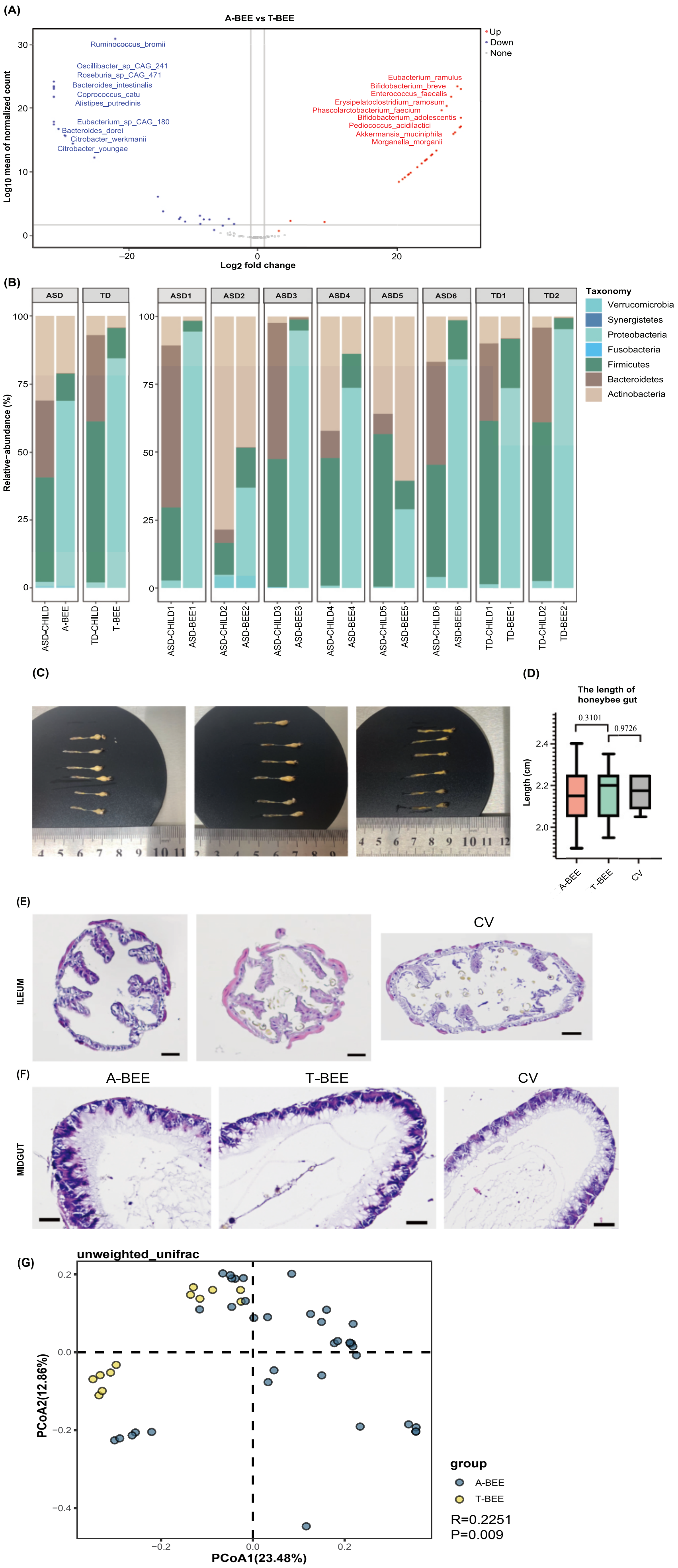
(A)
A-BEE vs T-BEE
Up
Down
None
Ruminococcus_bromii
Oscillibacter_sp_CAG_241
Roseburia_sp_CAG_471
Bacteroides_intestinalis
Coprococcus_catu
Alistipes_putredinis
Eubacterium_sp_CAG_180
Bacteroides_dorei
Citrobacter_werkmanii
Citrobacter_youngae
Eubacterium_ramulus
Bifidobacterium_breve
Enterococcus_faecalis
Erysipelatoclostridium_ramosum
Phascolarctobacterium_faecium
Bifidobacterium_adolescentis
Pediococcus_acidilactici
Akkermansia_muciniphila
Morganella_morganii
Log10 mean of normalized count
Log2 fold change
(B)
ASD
TD
ASD1
ASD2
ASD3
ASD4
ASD5
ASD6
TD1
TD2
Relative-abundance (%)
Taxonomy
Verrucomicrobia
Synergistetes
Proteobacteria
Fusobacteria
Firmicutes
Bacteroidetes
Actinobacteria
ASD-CHILD
A-BEE
TD-CHILD
T-BEE
ASD-CHILD1
ASD-BEE1
ASD-CHILD2
ASD-BEE2
ASD-CHILD3
ASD-BEE3
ASD-CHILD4
ASD-BEE4
ASD-CHILD5
ASD-BEE5
ASD-CHILD6
ASD-BEE6
TD-CHILD1
TD-BEE1
TD-CHILD2
TD-BEE2
(C)
(D)
The length of honeybee gut
0.3101
0.9726
Length (cm)
A-BEE
T-BEE
CV
(E)
ILEUM
CV
(F)
A-BEE
T-BEE
CV
MIDGUT
(G)
unweighted_unifrac
PCoA2(12.86%)
PCoA1(23.48%)
group
A-BEE
T-BEE
R=0.2251
P=0.009

Supplementary Figure 1. Effects of donor fecal microbiota colonization on the gut of honeybees.

(A) Volcano plot showing differences in species between groups. The differences were tested using DESeq2.

(B) Percentages of phyla in terms of abundance in the honeybee gut microbiota of the T-BEE and A-BEE group, and the abundance of phyla in the TD-CHILD and ASD-CHILD groups. Percentage of phyla in terms of abundance in honeybee gut microbiota of the A-BEE group (ASD-BEE 1-6) and T-BEE group (TD-BEE 1 and TD-BEE 2), and the abundance of phyla in the ASD-CHILD group (ASD-CHILD 1-6) and TD-CHILD group (TD-CHILD 1 and TD-CHILD 2).

(C) Images of honeybee intestines from each group.

(D) Length of honeybee intestines in each group. Group differences were tested using an unpaired t test (ns: $P > 0.05$, not significant).

(E-F) Histopathologic evaluation with hematoxylin-eosin staining showing structural disruption in the epithelium of ileum and midgut in each group (scale bars = 50 um).

(G) PCoA of unweighted UniFrac distances at the species level from the honeybee gut microbiota profiles of the A-BEE and the T-BEE groups. Differences between the groups were evaluated using PERMANOVA.

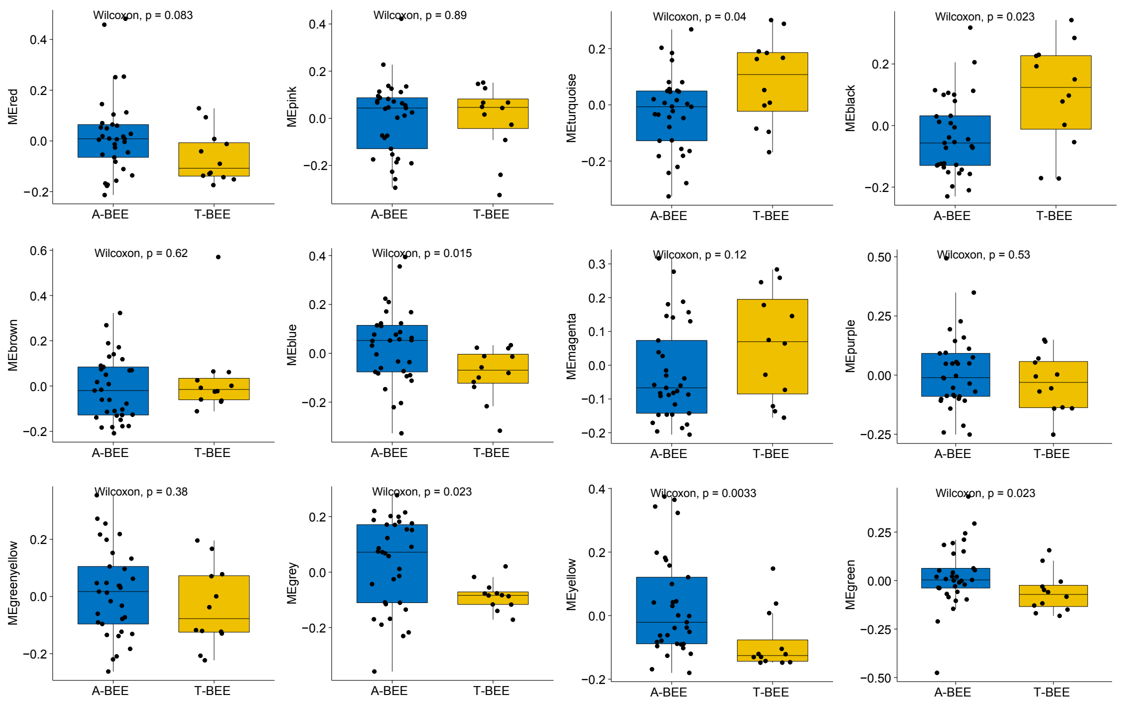

Supplementary Figure 2. Box plot showing the differences in the different modules between groups according to WGCNA.

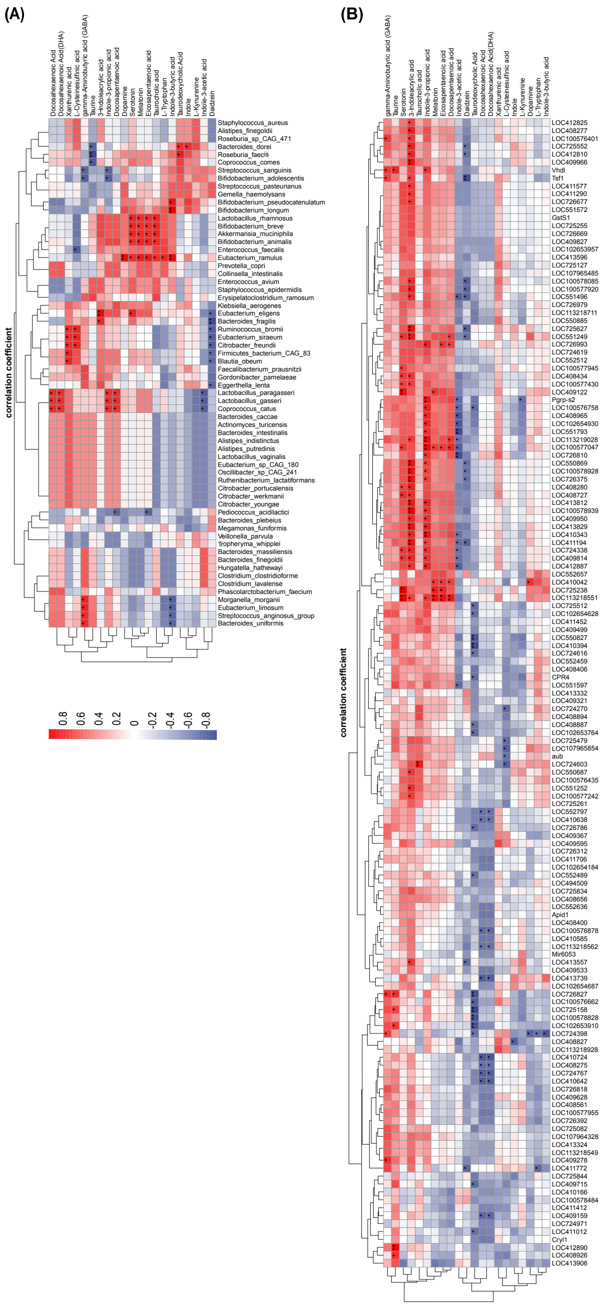

Supplementary Figure 3. Heat maps show the relationship between differential and species and metabolites, and the relationship between the MEblack module and metabolites.

(A)Heat maps showing the relationship between differential species and metabolites of interest, determined using a Spearman rank test. Heat map colors represent positive/negative Spearman correlation coefficients (*P < 0.05; **P < 0.01).

(B)Spearman rank test was used to determine this heat map showing the relationship between genes in the MEblack module and metabolites of interest. Color of the thermogram represents positive/negative Spearman correlation coefficients (*P < 0.05; **P < 0.01).

Supplementary Table 1. Basic information of donors.

Donor ID		TD-1	TD-2	ASD-1	ASD-2	ASD-3	ASD-4	ASD-5	ASD-6
Group		TD	TD	ASD	ASD	ASD	ASD	ASD	ASD
Sex		F	M	F	M	F	M	F	F
Age(year)		5	5	4	5	6	6	9	3
BMI（kg/m2）		16.36	14.6	15.4	16.3	14.4	15.1	16.2	17
History of high-dose antibiotics		NA	NA	NA	NA	NA	NA	NA	NA
Diagnostic age of ASD (year)		NA	NA	3	4	5	6	5	3
Routine treatments	Behavior therapy	NA	NA	ABA	ABA	ABA	ABA	ABA	ABA
	History of psychiatric medications	NA	NA	NA	NA	NA	NA	NA	NA
GI score		0	0	3	6	5	4	5	4
ADOS	Communication Sub	NA	NA	8	8	6	6	5	6
	Social sub	NA	NA	11	10	9	7	12	8
	Comm + Social	NA	NA	19	18	15	13	17	13
	Play/ imagination creativity total	NA	NA	4	4	4	4	4	4
	Stereo behav/ restric Interests	NA	NA	0	3	3	4	5	5
CARS-2 total		NA	NA	52	39	41	38.5	52	37
CARS-2	Relationship to people	NA	NA	4	4	4	3	4	3
	Imitation	NA	NA	4	4	3	3	4	3
	Remotional reponse	NA	NA	4	3	3	3	4	2
	Body use	NA	NA	3	2	2	3	3	3
	Object use	NA	NA	3	2	3	2.5	4	2.5
	Adaptation to change	NA	NA	2	2	3	2	4	2
	Visual response	NA	NA	4	3	2	2	4	3
	Listening response	NA	NA	4	2	2	2	3	2.5
	Taste-smell-touch response and use	NA	NA	4	2	3	3	3	1.5
	Fear and nervousness	NA	NA	1	1	1	1	0	1
	Verbal communication	NA	NA	4	4	4	3.5	4	4
	Non-verbal communication	NA	NA	3	3	4	2	3	3
	Activity level	NA	NA	4	2	2	1.5	4	2
	Level and consistency of intellectual response	NA	NA	4	2	1	3.5	4	1.5
	General impressions	NA	NA	4	3	4	3.5	4	3
Grade		NA	NA	severe	severe	severe	severe	severe	severe
